# Moderate prenatal stress may buffer the impact of Superstorm Sandy on placental genes: Stress in Pregnancy (SIP) Study

**DOI:** 10.1371/journal.pone.0226605

**Published:** 2020-01-29

**Authors:** Wei Zhang, Jacob Ham, Qian Li, Maya A. Deyssenroth, Luca Lambertini, Yonglin Huang, Kenji J. Tsuchiya, Jia Chen, Yoko Nomura

**Affiliations:** 1 Department of Psychology, Queens College, CUNY, New York, NY, United States of America; 2 Department of Psychology, New Jersey City University, Jersey City, NJ, United States of America; 3 Department of Psychiatry, Icahn School of Medicine at Mount Sinai, New York, NY, United States of America; 4 Department of Environmental Medicine and Public Health, Icahn School of Medicine at Mount Sinai, New York, NY, United States of America; 5 Department of Medicine, Endocrinology, Diabetes and Bone Disease, Icahn School of Medicine at Mount Sinai, New York, NY, United States of America; 6 Department of Obstetrics, Gynecology and Reproductive Science, Icahn School of Medicine at Mount Sinai, New York, NY, United States of America; 7 Department of Psychology, The Graduate Center, CUNY, New York, NY, United States of America; 8 Research Center for Child Mental Development, Hamamatsu University School of Medicine, Shizuoka, Japan; University of Insubria, ITALY

## Abstract

The placenta plays a central role in the epigenetic programming of neurodevelopment by prenatal stress (PS), but this pathway is not fully understood. It difficult to study in humans because the conditions for intense, traumatic PS are almost impossible to create ethically. This study was able to capitalize on a 2012 disaster that hit New York, Superstorm Sandy, to examine the impact of traumatic stress on placental gene expression while also examining normative PS, and compare the two. Of the 303 expectant mothers participating in the Stress in Pregnancy Study, 95 women were pregnant when Superstorm Sandy struck. During their pregnancy, participants completed self-report measures of PS and distress that were combined, using latent profile analysis, into one global indicator of normative PS. Placental tissue was collected at delivery and frozen for storage. RNA expression was assessed for 40 placental genes known to associate with the stress response system and neurodevelopment in offspring. Results showed that normative PS increased expression of just *MECP2*, *HSD11B2*, *and ZNF507*, whereas Superstorm Sandy PS decreased expression of *CDKL5*, *CFL1*, *DYRK1A*, *HSD11B2*, *MAOA*, *MAOB*, *NCOR1*, and *ZNF507*. Interaction analyses indicated that Superstorm Sandy PS was associated with decreased gene expression for the low and high PS group for *CFL1*, *DYRK1A*, *HSD11B2*, *MAOA*, and *NCOR1* and increased expression for the moderate PS group for *FOXP1*, *NR3C1*, and *NR3C2*. This study supports the idea that a moderate amount of normative PS may buffer the impact of traumatic PS, in this case caused by Superstorm Sandy, on placental gene expression, which suggests that the placenta itself mirrors the organism’s ability to develop an epigenetic resilience to, and inoculation from, stress.

## Introduction

Prenatal stress (PS) has been shown to impact offspring development over the lifespan [1–5]. Psychiatrically, PS increases the risk of behavioral and attentional disorders, autism and schizophrenia in male offspring and later-onset anxiety and affective disorders in females [3,6]. The biological impact of PS can include dysregulation of the hypothalamic pituitary adrenal (HPA) axis, broad alterations in brain growth, size or density, specific alterations to functional brain regions or neural components such as white matter abnormalities and hypomyelination in the hippocampus, prefrontal cortex, amygdala, and hypothalamus [7–13].

One of the biological pathways underlying this fetal programming is through the placenta, a maternal and fetal endocrine organ and the sole transporter and filter for nutrients, waste and teratogens. PS can have a direct impact on the placenta itself by altering its development especially early in pregnancy, changing structures that can lead to vasoconstriction of placental arteries or altering gene expression encoding for important functional proteins [4]. The epigenetic regulation of placental gene expression has been extensively explored in recent research, examining mechanisms such as DNA methylation, microRNA molecules, and histone modification [14–17].

One set of genes of great interest in the placental transmission of PS have been those involved in placental regulation of cortisol, the central stress hormone, and certain stress-related neurotransmitters [18]. The genes with the most substantive human research support are *NR3C1*, *HSD11B2*, *MAOA*, and *SLC6A4*. *HSD11B2* converts cortisol into its inactive form, cortisone. Cortisone is extremely important because fetal over-exposure to cortisol can lead to growth restriction, premature maturation of proliferative neural precursors, pre-term birth and altered HPA-axis development [18,19]. *NR3C1* encodes the glucocorticoid receptor that binds to cortisol [20]. In the placenta, it is postulated to be an upstream regulator of placental *HSD11B2* [21]. Methylation of *NR3C1* has been associated with a reactive, poorly regulated neurobehavioral profile in newborns [22] and behavior disorders in childhood [23]. *MAOA* metabolizes stress-related neurotransmitters such as serotonin and norepinephrine [24], and *SLC6A4* encodes the serotonin transporter [25]: over-exposure to stress-related neurotransmitters can have a significant impact on fetal development, synaptogenesis and neuronal cell division [26,27] and increase the risk for autism [28] and Attention Deficit /Hyperactivity Disorder (ADHD) [29].

PS can have an impact on the expression of placental genes, though the direction of impact varies for different genes and may depend on whether PS is measured as depression, anxiety or stress [2]. For the neurotransmitter genes, *MAOA* expression was found to decrease with greater maternal depression, whereas, *SLC6A4* expression increased with depression and anxiety [30,31]. Research on HPA-axis genes has produced even more complex findings. *NR3C1* increased with depression [32] but decreased with war trauma and normative stress [33]. *HSD11B2* has generally been found to decrease with normative stress, perceived stress and anxiety [34–36], though there are also a few studies reporting an increase in *HSD11B2*, but in response to traumatic stress [37,38].

Given the ethical limitations to stress research in humans, researchers have to capitalize where possible on natural disasters as proxies for the experimental introduction of traumatic PS. The largest natural disaster studied is the Quebec Ice Storm in 1998 [39,40]. There, researchers differentiated the objective impact of the storm in terms of loss, injury and physical impact from subjective perceptions of distress. Their results suggest that while objective and subjective PS are correlated, objective PS is associated with subsequent cognitive, linguistic and physical developmental outcomes [41–44], while subjective PS is associated with childhood anxiety, depression and aggression [40]. A study of the 2005 hurricane in New Orleans, Katrina, found that neither objective nor subjective PS predicted difficult infant temperament, but prenatal maternal mental health did [45]. A study of the 2008 flood in Iowa found that objective and subjective PS were related to cortisol reactivity in female toddlers alone [46]. Of note, none of these disaster studies investigated placental genomics, which we are suggesting may help us to understand the underlying mechanisms.

To advance our understanding of how PS impacts the placenta, we measured normative PS as maternal depression, anxiety, and lifetime histories of negative life events and traumatic Superstorm Sandy PS as prenatal exposure to Superstorm Sandy of 2012—the most destructive hurricane to ever strike New York City and at 50 billion dollars in damages, the second costliest natural disaster in the United States up to that point [47]. The impact in New York included 53 deaths, 305,000 homes destroyed, 250,000 vehicles damaged, massive power outages, flooding, and major disruption to the transit system [48]. We hypothesized that both normative and traumatic PS would impact placental gene expression, though the direction of impact would depend on type of PS and type of gene. We also explored whether normative PS might alter the impact of traumatic Superstorm Sandy PS on placental gene expression.

## Materials and methods

### Participants

Participants came from the Stress in Pregnancy (SIP) Study [49]https://paperpile.com/c/rh5zV9/EFym, an ongoing longitudinal study begun in 2009, that examines the impact of PS on child neurodevelopment. Expectant mothers in the second trimester were recruited from obstetrics clinics at Mount Sinai Hospital and New York-Presbyterian/Queens in New York City. Women were excluded based on HIV infection, maternal psychosis, maternal age < 15 years, life-threatening maternal medical complications, and congenital or chromosomal abnormalities in the fetus. Written informed consent was obtained in all cases. The Institutional Review Boards at the City University of New York, Icahn School of Medicine at Mount Sinai, and New York Presbyterian/Queens approved the study.

Superstorm Sandy hit New York City in October 2012, affecting 408 SIP Study families, of which 303 had complete data for this study. These 303 did not differ demographically from the full cohort. Among the 303, 95 dyads experienced the storm during pregnancy.

The mean (±standard deviation) age of mothers was 27 (±6.0) years. The mothers were Hispanic/Latino (53%), Black (24%), White (9%), Asian (8%) and other (6%). Though 58% of mothers attended college, only 18% had completed a bachelor or graduate degree. A small majority of mothers were single (57%), while 40% were married or in a common law marriage. Among offspring, 52% were male.

## Exposure to superstorm sandy

The specific gestational timing during which Superstorm Sandy occurred was calculated based on the date of birth of the child and the day the storm hit the metropolitan New York area (October 29, 2012), and serves as our primary measure of exposure to Superstorm Sandy. Following the classification, a dichotomous variable was created to categorize mothers as either pregnant during Superstorm Sandy (Exposed, n = 95) or pregnant before or after the storm (Non-Exposed, n = 208). Of the 95 exposed, 66 participants experienced the storm during the first trimester and 29 during the 2nd or 3rd trimesters.

### Normative prenatal stress

Mothers completed the following five self-report scales of normative PS during the second trimester. The 10-item Edinburgh Postnatal Depression Scale (EPDS) rates the severity of depressive symptoms from 0 to 3. Items were summed, with scores above 13 suggesting clinical levels of depression [50]. The inventory is well-validated in different languages and has acceptable reliability, sensitivity and specificity [51]. The 10-item Pregnancy Related Anxieties Questionnaire-Revised (PRAQ-R) measures pregnancy related fears and worries, rated from 1 (definitely not true) to 5 (definitely true) [52], with three subscales: fear of giving birth, fear of bearing a handicapped child, and concerns about changes in appearance. Subscale scores are an average of item scores, and the total score, ranging from 3 to 15, is the sum of subscale scores. PRAQ-R has good reliability and validity in predicting adverse child behaviors and developmental delays [53]. The 40-item State-Trait Anxiety Inventory (STAI) assesses temporary “state anxiety” and long-standing, characterological “trait anxiety” [54]. Each subscale consists of 20 items rated from 1 to 4, summed to produce subscale scores ranging from 20 to 80. In one normative sample, working females aged 19–39 reported average subscale scores of 36.17 (*SD* = 10.96) [55]. A meta-analysis found the STAI to have very good internal consistency [56]. The 14-item Perceived Stress Scale (PSS-14) assesses how often raters appraise situations in the past month as stressful from 0 to 4 [57]. The total score was computed by reverse scoring positively stated items and then summing the scores. Total scores ranged from 0 to 56. PSS-14 has adequate internal consistency and test-retest reliability [57,58]. The 23-item Psychiatric Epidemiology Research Interview Life Events Scale (PERI LES) [59] has widely been used to study the effects of adverse life events during pregnancy [60–62]. PERI LES assesses five major areas of life: relationships, health, legal matters, work and finances, and friendships. Participants further categorize the stressors as either a “good” or “bad” experience, the total number of which represents the positive and negative scales. The LES has been validated against narrative reports of life events [63].

The six PS measures were amalgamated into one normative PS scale, because (1) they were all significantly correlated (*r*_*s*_ = .18 to .83; *p* < .01; S1 Table), (2) they were always correlated in the same direction with individual gene expression levels (S1 Table), and (3) other researchers have amalgamated maternal distress variables in prior research [64–66]. The amalgamated PS scale was created using latent profile analysis (LPA) with MPlus. Missing data were negligible (EPDS, 2.3%; PRAQ-R, 3%; PSS-14, 0.7%; STAI, 2.3%; and negative PERI LES, 1%), and were imputed using full maximum likelihood estimation. LPA produced models with two to four levels. The three-level model, representing Low, Moderate, and High PS, best fit the data based on Bayesian Information Criteria [67], adjusted BIC [68], Lo-Mendell-Rubin test [69] and entropy values (S2 Table). Sample sizes for each PS group were 116 for Low, 132 for Moderate and 55 for High PS.

### Placenta collection and gene expression profiling

At delivery, research staff gathered medical birth records and collected placentas. Placenta biopsies, free of maternal decidua, were collected from each quadrant midway between the cord insertion and the placenta rim within one hour of delivery to prevent RNA degradation. The placentas were snap-frozen in liquid nitrogen and stored at -80°C. RNA was extracted with the Maxwell 16 automated DNA/RNA extraction equipment (Promega: Madison, WI) using the proprietary extraction kits following the manufacturer’s protocol. RNA was quantified with Nanodrop spectrophotometer (Thermo Electron North America: Madison, WI).

Forty candidate genes were identified *a priori* for their involvement in HPA-axis functioning and neurodevelopment, based on an extensive literature search and using the Ingenuity® Knowledge Base (http://www.ingenuity.com). Placental RNA was profiled using nCounter by nanoString Technologies (Seattle, WA) as described elsewhere [70,71]. Nanostring data were normalized using the NanoString Norm package [72]. First, raw code counts were normalized against the geometric mean of spike-in controls to account for differences in hybridization and recovery. Differences in sample content were accounted for by normalizing the data against the geometric mean of housekeeping genes (*GAPDH*, *RPL19*, and *RPLP0*). The background threshold was set to the limit of detection divided by the square root of two to maintain sample variability. Thirteen genes were considered unexpressed and omitted from analysis because more than 50% of the sample fell below the limit of detection. Of the remaining 27 genes, 14 genes were related to HPA-axis function, and 13 genes were related to neurodevelopment (S3 Table).

### Statistical analyses and covariates

Differences across demographic variables among the PS groups were examined using ANOVA for continuous variables and Chi-square/Fisher's exact tests for categorical variables. The impact of normative and traumatic PS, and their interaction, on placental gene expression was assessed using a general linear model (GLM), controlling for infant gender, maternal race and education, and delivery mode, selected based on prior research [24,73,74] (significance set at *p* < 0.05). In order to control for Type I errors due to multiple testing, we made an adjustment using the Benjamini–Hochberg procedure [75,76].

## Results

### Descriptive analyses

Differences between PS groups were examined using descriptive analyses on demographic and stress variables. As shown in Table 1, the PS groups did not differ on maternal demographic characteristics, but there were relatively more female infants in the Moderate PS group (*p* = .008). Table 2 shows the differences between Superstorm Sandy and the control groups in birthweight, race, marital status, and education levels. Means and SDs for all stress measures by PS groups are listed in Table 3. Based on commonly used clinical norms available for the EPDS and STAI, the High PS group was above the clinical cutoff for depression on the EPDS and in the Moderate Anxiety range for State Anxiety, which provides an informal validation of the classification.

**Table 1:** Demographic characteristics of participants in total and by normative prenatal stress groups.

		Total Sample		Low PS		Moderate PS		High PS		*p* value
		(n = 303)	(n = 116)	(n = 132)	(n = 55)	
**Infant sex**										0.008
Males	N (%)	158	(52%)	64	(55%)	57	(43%)	37	(67%)	
Females	N (%)	145	(48%)	52	(45%)	75	(57%)	18	(33%)	
**Gestational age (wks)**	Mean (SD)	39.1	(2.07)	39.2	(2.22)	39.04	(2.02)	39.04	(1.89)	0.8
**Birthweight (g)**	Mean (SD)	3268	(594)	3308	(643)	3255	(574)	3211	(536)	0.6
**Maternal race**										0.2
White	N (%)	27	(9%)	7	(6%)	13	(10%)	7	(13%)	
Black	N (%)	74	(24%)	32	(28%)	35	(27%)	7	(13%)	
Hispanic/Latino	N (%)	159	(52%)	64	(55%)	61	(46%)	34	(62%)	
Asian	N (%)	23	(8%)	6	(5%)	12	(9%)	5	(9%)	
Others	N (%)	18	(6%)	7	(6%)	9	(7%)	2	(4%)	
Missing						2	(1%)			
**Maternal education**										0.7
Primary school	N (%)	6	(2%)	2	(2%)	2	(2%)	2	(4%)	
Some high school	N (%)	53	(17%)	22	(19%)	20	(15%)	11	(20%)	
High school graduate	N (%)	67	(22%)	25	(22%)	35	(27%)	8	(15%)	
Some college	N (%)	91	(30%)	39	(34%)	36	(27%)	16	(29%)	
Associate degree	N (%)	30	(10%)	8	(7%)	15	(11%)	7	(13%)	
Bachelor’s degree	N (%)	30	(10%)	9	(8%)	15	(11%)	6	(11%)	
Graduate degree	N (%)	25	(8%)	11	(9%)	9	(7%)	5	(9%)	
**Marital status**										0.7
Married	N (%)	101	(33%)	37	(32%)	45	(34%)	19	(35%)	
Common law	N (%)	21	(7%)	6	(5%)	12	(9%)	3	(5%)	
Single	N (%)	174	(57%)	71	(61%)	71	(54%)	32	(58%)	
Widowed	N (%)	2	(1%)	0	(0%)	1	(1%)	1	(2%)	
Divorced/separated	N (%)	3	(1%)	1	(1%)	2	(2%)	0	(0%)	
Missing	N (%)			1	(1%)	1	(1%)			

**Table 2:** Demographic characteristics of participants by sandy prenatal stress groups.

		No Sandy		Sandy		
		PS		PS		*p* value
		(n = 208)		(n = 95)		
**Infant sex**						0.542
Males	N (%)	106	(51%)	52	(55%)	
Females	N (%)	102	(49%)	43	(45%)	
**Gestational age (wks)**	Mean (SD)	39.05	(2.23)	39.22	(1.69)	0.5
**Birthweight (g)**	Mean (SD)	3202.44	(620.78)	3408.69	(507.09)	0.003
**Maternal race**						0.015
White	N (%)	15	(7%)	12	(13%)	
Black	N (%)	58	(28%)	16	(17%)	
Hispanic/Latino	N (%)	111	(53%)	48	(51%)	
Asian	N (%)	10	(5%)	13	(14%)	
Others	N (%)	13	(6%)	5	(5%)	
Missing		1	(~0%)	1	(1%)	
**Maternal education**						< .001
Primary school	N (%)	5	(2%)	1	(1%)	
Some high school	N (%)	47	(23%)	6	(6%)	
High school graduate	N (%)	48	(23%)	20	(21%)	
Some college	N (%)	66	(32%)	25	(26%)	
Associate degree	N (%)	16	(8%)	14	(15%)	
Bachelor’s degree	N (%)	14	(7%)	16	(17%)	
Graduate degree	N (%)	12	(6%)	13	(14%)	
**Marital status**						< .001
Married	N (%)	50	(24%)	51	(54%)	
Common law	N (%)	14	(7%)	7	(7%)	
Single	N (%)	140	(67%)	34	(36%)	
Widowed	N (%)	2	(1%)	0	(0%)	
Divorced/separated	N (%)	1	(~0%)	2	(2%)	
Missing	N (%)	1	(~0%)	1	(1%)	
**Normative Stress**	Mean (SD)	1.8	(0.74)	1.79	(0.70)	0.882

**Table 3:** Mean scores on individual normative prenatal stress (ps) measures among total sample and ps levels identified through latent profile analysis.

	Total		Low PS		Moderate PS		High PS	
	(N = 303)		(n = 116)		(n = 132)		(n = 55)	
	*M*	*(SD)*	*M*	*(SD)*	*M*	*(SD)*	*M*	*(SD)*
Prenatal depression (EPDS)	7.36	(5.40)	3.02	(3.13)	7.87	(3.43)	15.09	(3.22)
Pregnancy-related anxiety (PRAQ-R)	5.86	(2.29)	4.69	(1.65)	6.03	(1.95)	7.94	(2.65)
Perceived prenatal stress (PSS-14)	36.39	(7.38)	31.07	(6.35)	37.85	(5.32)	44.02	(4.93)
State anxiety (STAI-S)	37.94	(11.59)	27.13	(4.97)	40.94	(6.72)	53.65	(7.88)
Trait anxiety (STAI-T)	38.39	(10.76)	27.97	(4.74)	41.03	(4.98)	54.13	(5.83)
Number of negative stressful life event (LES)	1.57	(2.02)	0.91	(1.50)	1.39	(1.54)	3.38	(2.81)

### Associations between Superstorm Sandy exposure and placental gene expression

**Fig 1:**
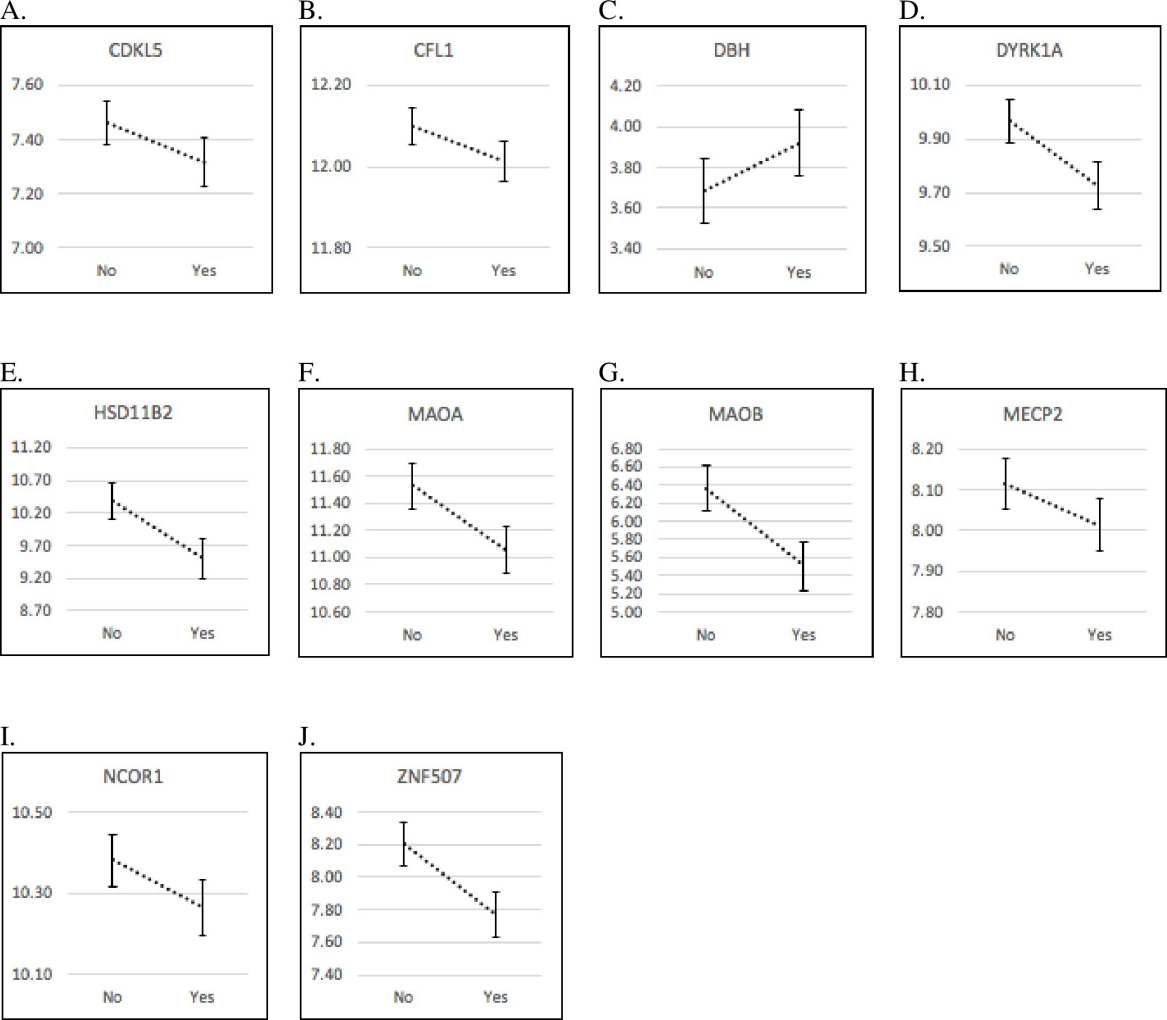
Marginal Mean (SE) Gene Expression Level (represented on the Y axis) by Traumatic Prenatal Stress (yes = Storm Exposure, no = No Storm Exposure). (A) *CDKL5* gene expression by traumatic prenatal stress. (B) *CFL1* gene expression by traumatic prenatal stress. (C) *DBH* gene expression by traumatic prenatal stress. (D) *DYRK1A* gene expression by traumatic prenatal stress. (E) *HSD11B2* gene expression by traumatic prenatal stress. (F) *MAOA* gene expression by traumatic prenatal stress. (G) *MAOB* gene expression by traumatic prenatal stress. (H) *MECP2* gene expression by traumatic prenatal stress. (I) *NCOR1* gene expression by traumatic prenatal stress. (J) *ZNF507* gene expression by traumatic prenatal stress.

Table 4 shows genes that were significantly or marginally significantly associated with the main effects of normative, Superstorm Sandy PS, or their interactions. Prenatal storm exposure significantly or marginally significantly predicted decreased expression of *CDKL5* (*p* = .053), *CFL1* (*p* = .046), *DYRK1A* (*p* = .002), *HSD11B2* (*p* < .001), *MAOA* (*p* = .002), *MAOB* (*p* < .001), *MECP2* (*p* = .056), *NCOR1* (*p* = .052), and *ZNF507* (*p* < .001). The one exception, *DBH* was related to a marginally significant *increase* in expression with storm exposure (*p* = .076). Graphs of the mean expression levels of each gene are presented in Fig 1A–1J. The results were calculated using the GLM models. Not all genes expressed in the sample. In the sections regarding PS and placental gene expression, we did not describe all non-significant associations. Reported p-values are FDR-adjusted.

**Table 4:** Significant p-values (FDR-adjusted p-values) in the Prediction of Gene Expression using Normative Prenatal Stress (PS), Superstorm Sandy PS and their Interaction: GLM Model.

Gene	Normative PS Main Effect	Normative PS Quadratic	Superstorm Sandy PS Main Effect	Normative x Superstorm Sandy
*CDKL5*			0.042 (0.053)	0.095 (0.10)
*CFL1*			0.028 (0.046)	0.026 (0.053)
*CRHBP*	0.014 (0.04)	0.007 (0.007)		
*DBH*			0.076 (0.076)	
*DYRK1A*			0.001 (0.002)	0.012 (.042)
*FOXP1*		0.071 (0.077)		0.011 (.042)
*HSD11Β2*	0.099 (0.099)		< 0.001 (<0.001)	0.049 (0.057)
*MAOA*			0.001 (0.002)	0.085 (0.095)
*MAOB*			< 0.001 (<0.001)	
*MECP2*	0.046 (0.090)		0.051 (0.056)	0.09 (0.095)
*NCOR1*			0.039 (0.052)	0.083 (0.095)
*NR3C1*		0.077 (0.077)		0.036 (0.076)
*NR3C2*				0.038 (0.076)
*ZNF507*	0.068 (0.092)		< 0.001 (< .001)	

Note: P-values are calculated based on GLM controlling for infant gender, maternal race and education, and delivery mode. Values in the parentheses are the FDR-adjusted p-values

### Association between PS and placental gene expression

**Fig 2:**
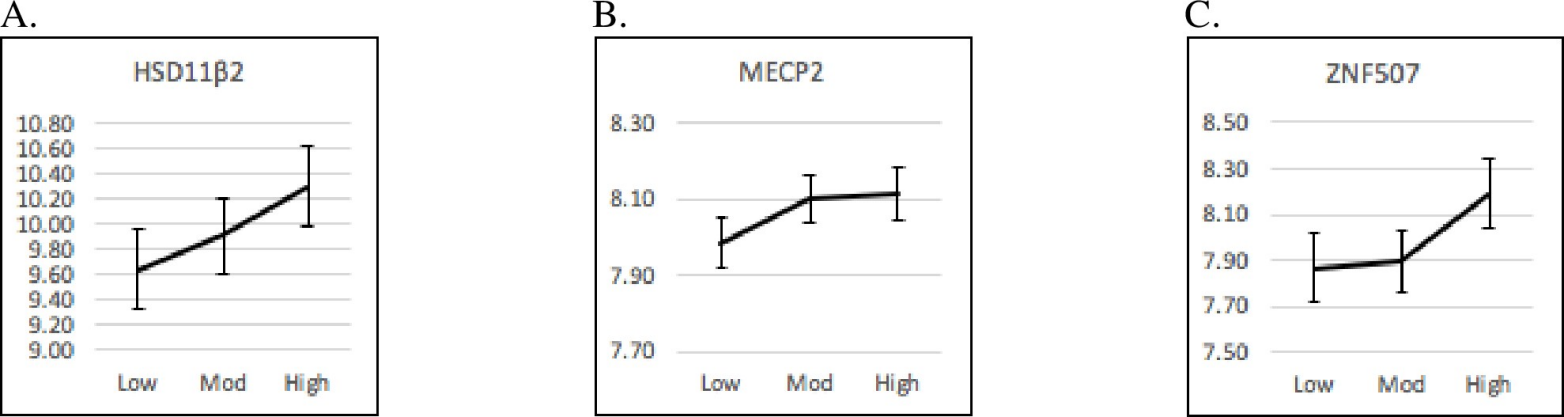
Marginal Mean (SE) Gene Expression Levels (represented on the Y axis) by Normative Prenatal Stress (PS): Linear Patterns (Low = low PS; Mod = moderate PS; High = high PS). (A) *HSD11B2* gene expression levels by normative prenatal stress: linear pattern. (B) *MECP2* gene expression levels by normative prenatal stress: linear pattern. (C) *ZNF507* gene expression levels by normative prenatal stress: linear pattern.

The overall group difference in normative PS levels was shown to be marginally significantly and positively related to *MECP2* (p = .090) using GLM. Pairwise comparisons of means showed mean gene expression levels for Moderate PS were marginally greater than for Low PS (p = .073). A similar, though only marginally significant, pattern in overall group differences was found for *HSD11B2* (p = .099) and *ZNF507* (p = .092). Pairwise comparisons showed that the mean expression level of *HSD11B2 and ZNF507* was marginally greater for high PS versus low PS (p = .099, .099 respectively). Graphs of the means of gene expression are presented in Fig 2A–2C.

**Fig 3:**
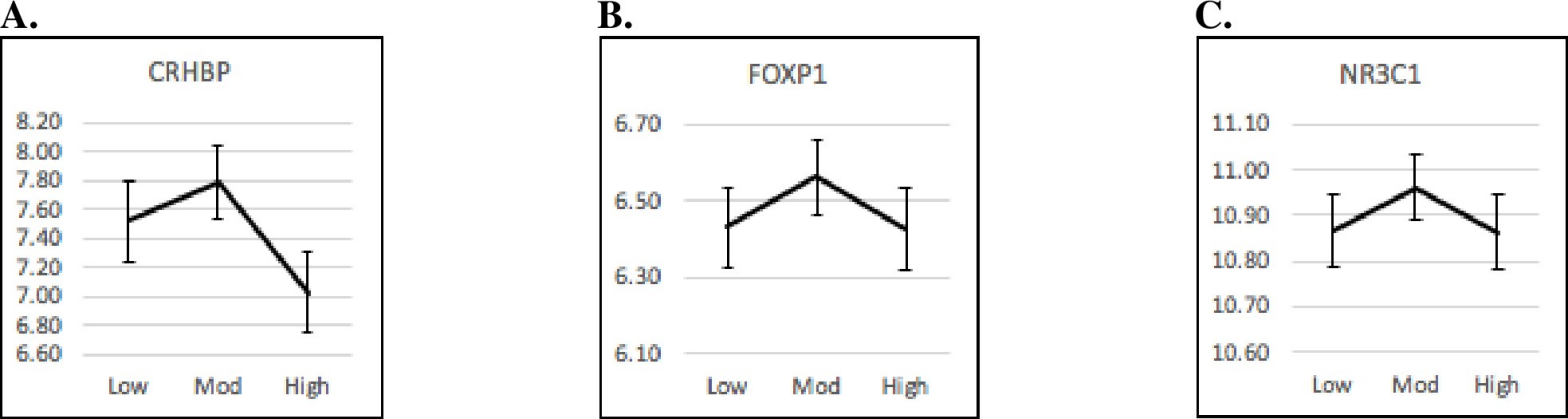
Marginal Mean (SE) Gene Expression Levels (represented on the Y axis) by Normative Prenatal Stress: Inverted-U Shaped Patterns (Low = low PS; Mod = moderate PS; High = high PS). (A) *CRHBP* gene expression levels by normative prenatal stress: Inverted-U shaped pattern. (B) *FOXP1* gene expression levels by normative prenatal stress: Inverted-U shaped pattern. (C) *NR3C1* gene expression levels by normative prenatal stress: Inverted-U shaped pattern.

One gene, *CRHBP*, was significantly related to PS (*p* = .007) in a curvilinear, inverted-U shape, when analyzing overall group differences. The marginal mean of *CRHBP* expression was significantly smaller for high PS compared to moderate PS (*p* = .011). *FOXP1* and *NR3C1* also showed similar, though only marginally significant, curvilinear patterns (*p* = .077 and .077 respectively). Fig 3A–3C shows the means for these curvilinear patterns.

### Differential impact of superstorm sandy PS by normative PS

**Fig 4:**
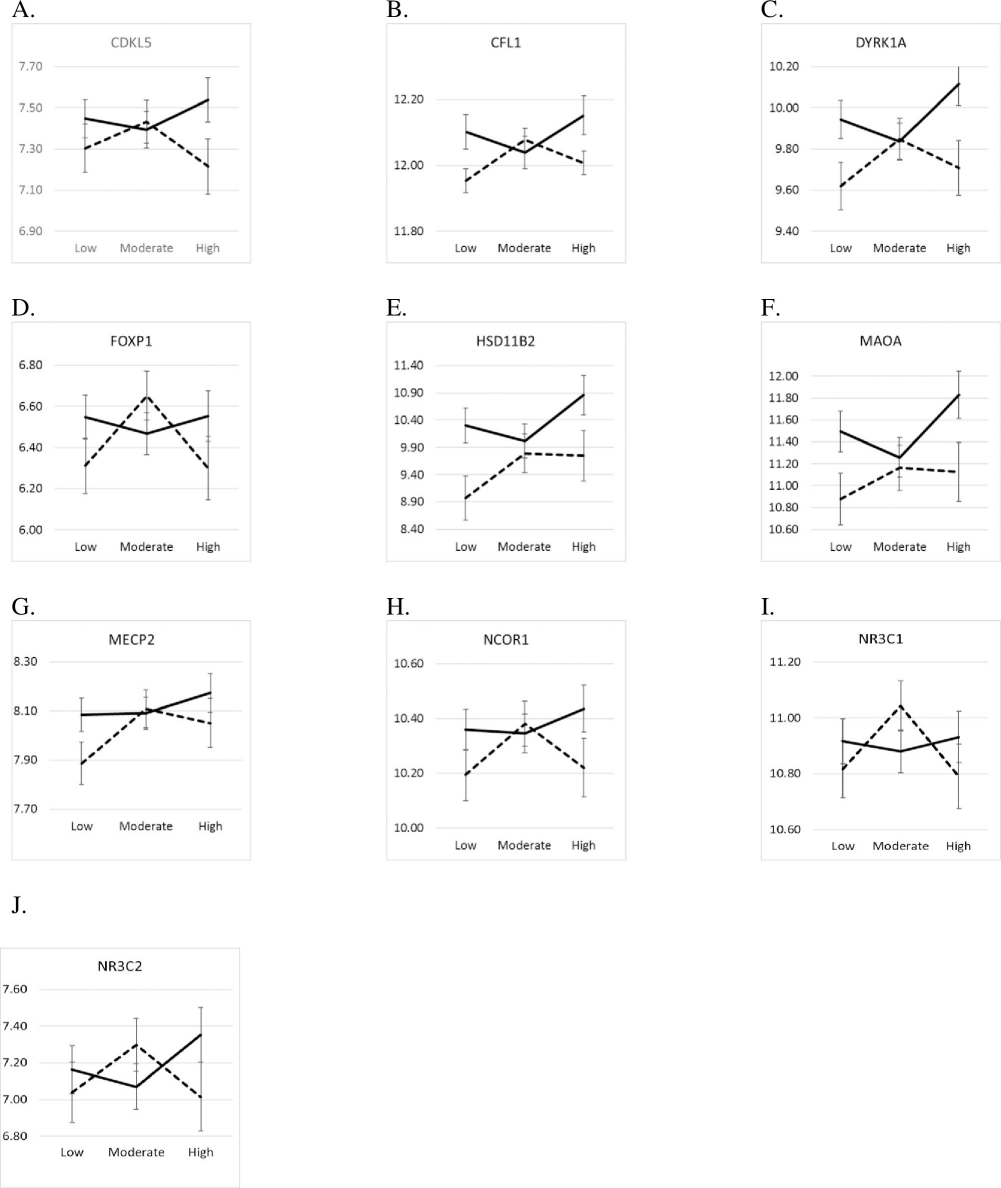
Marginal Mean (SE) Gene Expression Levels (represented on the Y axis) by Normative Prenatal Stress X Superstorm Sandy Prenatal Stress (Storm Exposure) (Solid Line = No Storm Exposure; Dotted Line = Storm Exposure) (Low = low PS; Mod = moderate PS; High = high PS). (A) *CDKL5* by normative prenatal stress X Superstorm Sandy prenatal stress. (B) *CFL1* by normative prenatal stress X Superstorm sandy prenatal stress. (C) *DYRK1A* by normative prenatal stress X Superstorm Sandy prenatal stress. (D) *FOXP1* by normative prenatal stress X Superstorm Sandy prenatal stress. (E) *HSD11B2* by normative prenatal stress X Superstorm Sandy prenatal stress. (F) *MAOA* by normative prenatal stress X Superstorm Sandy prenatal stress. (G) *MECP2* by normative prenatal stress X Superstorm Sandy prenatal stress. (H) *NCOR1* by normative prenatal stress X Superstorm Sandy prenatal stress. (I) *NR3C1* by normative prenatal stress X Superstorm Sandy prenatal stress. (J) *NR3C2* by normative prenatal stress X Superstorm Sandy prenatal stress.

To assess whether normative PS might moderate the impact of prenatal storm exposure, an interaction term for the two was included in the original GLM with normative PS, traumatic PS and the noted covariates. The most prevalent pattern of findings was where Superstorm Sandy PS was associated with decreased gene expression for the low and high PS groups but not Moderate PS. This pattern was significant or marginally significant for *CFL1* (*p* = .053), *DYRK1A* (*p* = .042), *FOXP1* (*p* = .042), *HSD11B2* (*p* = .057), *NR3C1* (*p* = .076), *NR3C2* (*p* = .076), *MAOA* (*p* = .095), and *NCOR1* (*p* = .095). While only marginal, the pattern was partially supported for *MECP2* (*p* = .095) and *CDKL5* (*p* = .10) in that expression level was low only for the High PS group. Fig 4A–4K show the means for the interaction effects.

## Discussion

In this study, we examined whether traumatic and/or normative PS impacted the expression of placental genes related to HPA-axis function and neurodevelopment. Further, we examined whether the impact of Superstorm Sandy PS depended on the level of normative PS. Normative PS was defined as depression, anxiety, pregnancy-related stress, and negative life events. Superstorm Sandy PS was defined as prenatal exposure to Superstorm Sandy.

Our findings presented a complex narrative for how normative and Superstorm Sandy PS might impact expression patterns of some, but not all, placental genes. Our result runs counter to other studies that found an increase in expression in response to PS [36]. First, in terms of main effects, Superstorm Sandy PS generally *downregulated* placental gene expression (*CDKL5*, *CFL1*, *DYRK1A*, *HSD11B2*, *MAOA*, *MAOB*, *NCOR1*, and *ZNF507*). Their downregulation suggests one pathway through which expression of other genes might be altered by stress from extreme events such as disasters.

The most interesting of these impacted genes is *HSD11B2*, which is centrally involved in prenatal stress because it buffers fetal exposure to cortisol. *MAOA* metabolizes stress-related neurotransmitters such as serotonin and norepinephrine [24]. *MAOB* regulates the stress-related neurotransmitter, dopamine, but is minimally present in the placenta [77]. *CFL1* and *DYRK1A* are involved in cell division and proliferation. *DYRK1A* has been linked to the memory and learning deficits associated with Down syndrome [78,79]. The remaining four genes (*CDKL5*, *MECP2*, *NCOR1*, and *ZNF507*) are transcription regulators. *CDKL5* and *MECP2* have been associated with Rett’s Disorder [80–82]. *ZNF507* has been implicated in schizophrenia [83].

In contrast to the large impact of Superstorm Sandy PS, normative PS alone only marginally impacted expression of *MECP2*, *ZNF507* and *HSD11B2*, all in the direction of increased expression, which is opposite to the impact of Superstorm Sandy PS. Notably, the *upregulation* of *HSD11B2* is opposite to the results of prior studies that showed PS downregulating *HSD11B2 e*xpression [34,36,84]. It may be that our definition of Superstorm Sandy PS was too general, in contrast to, for example, a very discrete event like an amniocentesis [85]. A natural disaster, especially including an extended aftermath, might be better be defined as an intense normative stressor. However, this explanation does not account for why the natural disaster would impact gene expression in the opposite direction to normative PS as we defined it in this study. Perhaps, the best conclusion is to be left wondering at the complex and dynamic ways in which stress in all its forms appears to impact the placenta rather than drawing simple, unidirectional and reductionistic conclusions.

Results from our interaction analysis suggest a framework of dynamic complexity. For five of the eight genes downregulated by Superstorm Sandy PS alone (*CFL1*, *DYRK1A*, *HSD11B2*, *MAOA*, and *NCOR1*), moderate normative PS appeared to nullify the effect of Superstorm Sandy PS such that only the Low or High normative PS groups showed significant reductions in gene expression associated with traumatic PS. *CDKL5* and *MECP2* showed a similar result though only the High or the Low normative PS group, respectively, showed significantly different estimated marginal means in gene expression. *FOXP1*, *NR3C1* and *NR3C2* showed a variant of the pattern in which the Moderate normative PS group showed a significant or marginally significant increase in gene expression. FOXP1 is a transcription repressor involved in brain function and neurodevelopment [86]. Altered levels of *FOXP1* expression have been linked to abnormal neurodevelopment and autism [87–89]. *NR3C1* and *NR3C2* produce the glucocorticoid and mineralocorticoid receptors, which bind to cortisol, and may increase sensitivity to glucocorticoids [18] and regulate expression of *HSD11B2* [21]. They are some of the most widely studied genes and generally have been found to decrease in expression in response to prenatal stress [90].

Finally, *CRHBP*, which regulates the stress response by binding to corticotropin-releasing hormone [91–94], was the one gene that produced a divergent but related pattern to the others. It was *downregulated* in response to normative PS but appeared to follow a curvilinear, inverted-U shaped pattern, resulting in the moderate normative PS group having the highest average gene expression levels. However, neither main effect of Superstorm Sandy PS nor the interaction with normative PS altered its expression.

The interaction analyses suggest that moderate, normative PS may protected he placenta from Superstorm Sandy PS; whereas, low normative PS left the placenta unprepared, and high normative PS may have overwhelmed the placenta’s tolerance for stress. The possibility that moderate normative PS might buffer the placenta from Superstorm Sandy PS was anticipated by various theories encapsulated under the Developmental Origins of Health and Disease hypothesis (DOHaD) [95], such as Allostatic Load [96], Predictive Adaptive Response [97] and the three-hit concept of vulnerability and resilience [98], which generally conceptualize epigenetic responses as anticipatory adaptations to an environment such as the one the mother experiences during pregnancy. In these models, PS signals an environment that is either impoverished or dangerous and thus programs offspring, especially male offspring, to grow to become smaller (to need less energy), slower in metabolism (to better conserve it), faster to mature and more vigilant, impulsive, aggressive and unemotional (to better fend off competitors and predators) [99]. Additionally, these DOHaD theories can be viewed as consonant with a stress inoculation model [100] in which moderate stress exposure protects the organism from future stress—a model consistent with our results.

### Limitations and future research

First, our hypotheses about how changes in gene expression operate in the placenta must be recognized mostly as speculation since the level of variance explained by normative and/or traumatic PS on placental gene expression was minimal (**Fig 1**). Several genes, especially *HSD11B2* and *SCL6A4*, have been extensively studied as they regulate maternal neurotransmitters, thus consequently affecting the developing fetus more directly, whereas a gene such as *MAOA* is less studied, requiring the present findings to be replicated in different studies. While our findings wait for replication and validation in other studies, we are planning to evaluate subsequent neurobehavioral outcomes in the offspring in relation to the current, complex findings of PS and placenta genes. It is our belief that this will further aid understanding the DOHaD hypotheses that involve genes censoring the prenatal environment and adaptation to the predicted postnatal environment for the best survival trajectory. These hypotheses need to be substantiated through more research including longitudinal studies on child development looking at epigenetic mechanisms. Our hypotheses are based on the known functions of the various genes in question and the function of the proteins they program. Further, although our total sample was relatively large, especially for an opportunistic study that capitalized on a natural disaster, the size of the high normative PS group was much smaller than the others (n = 55 for the High PS as compared to 132 for Moderate PS and 116 for Low). These sample size differences are unavoidable when looking for extremes of distress in a normative sample. Future studies with different community and clinical samples would help substantiate our findings. Another limitation of a natural disaster study is limited control of the timing of storm exposure– 66 of the 95 storm-exposed pregnancies came during the first trimester. Our current understanding, through previous studies such as the Dutch Cold Winter Study, broadly suggests that stress exposure across different trimesters give rise to different programming outcomes [101–103]. It is generally believed that exposure to stress during early pregnancy is often associated with greater risk for disrupted developmental programming and increased risk for neurodevelopmental disorders in offspring. Also, much of our original baseline data were collected during the second trimester, so storm exposure may have impacted ratings of normative PS before we measured normative PS. However, we found a very small correlation between storm exposure and normative PS classification. Further, although normative PS was measured primarily in the 2^nd^ trimester, current research suggests that normative PS ratings are highly consistent throughout pregnancy [32,104,105]. We are aware that we are examining placental gene expression at term, and therefore we are examining how PS throughout pregnancy interacts with a traumatic Superstorm Sandy stressor that may have occurred early in pregnancy, but whose residual impact may have continued for much longer. Recent research on the 2011 Queensland Flood revealed placental glucocorticoid and glucose systems gene expression were associated with natural disaster-related PS (i.e., objective hardship and subjective distress) [106]. One limitation of our study is that we only assessed exposure to the storm and not the more nuanced physical or psychological impact of the storm such as objective hardship, measured by Storm32 and developed by King and Laplante [107, 108] or subjective distress, measured by the Impact of Event Scale-Revised [109]. In order to explore whether the degree of traumatic exposure to Superstorm Sandy (either subjectively or objectively) was associated with gene expression levels in a dose response fashion, we conducted a *post-hoc* analysis using the placentas of the exposed (N = 95). In this *post-hoc* analysis, we found no notable associations between either objective hardship or subjective distress, with the exception of SRD5A3. SRD5A3 was upregulated as the degree of objective hardship (p = .03) and subjective distress (p = .04) increased. As our sample size for this subsample analysis was relatively low, future studies with a larger sample size should include a more elaborate measure of the subjective or objective impact of the storm, such as Project Ice Storm [39] managed, and the duration of impact. In addition, we did not investigated the role of pregnancy diseases such as endometriosis, pre-eclampsia, and intrauterine growth restriction, which may affect both reported stress and placenta epigenetics [16,110–113]. The restriction of our analyses on placentas from healthy pregnancies may have constrained variances in the stress impacts and placental gene expression. However, the addition of HPA axis functioning measures (e.g., maternal cortisol) would strengthen the present findings with the placental glucocorticoid genes.

The most important question is whether and how specific alterations in placental gene expression shape the lifelong development and functioning of the offspring and perhaps future generations. The adaption of placental gene expression in response to PS may be seen as mediating between PS and an increased propensity for future neurobehavioral dysregulation and disorders. This can be investigated by combing neurodevelopmental and biological assessments (e.g., brain imaging and cortisol measures) in the offspring. The pathway is certainly complex, nonlinear, and dynamic, and further shaped by interacting endogenous, exogenous, and epigenetic factors. In this study we focused on mRNA expression and did not measure epigenetic mechanisms that regulate gene expression (e.g., miRNAs, DNA methylation) [1,17,114]. Though the research has focused on the impact of placental genes on fetal programming, we recognize that the placenta plays a central role in priming maternal behavior as well [115] and acknowledge the existing research on the epigenetics of early child-rearing [1,4,88,98]. So far we believe that stress during early pregnancy may begin to shape the mother, the fetus and the placenta itself both phenotypically and epigenetically, which then primes but does not predestine the adaptation of the maternal-offspring complex to future environments and lead to further alterations in phenotypic and epigenetic function.

### Conclusion

Though DOHaD researchers have applied theories of predictive adaptability and stress inoculation to explain the impact of PS on fetal programming (96–98), to our knowledge this is the first study to document evidence in support of these theories in relation to the life of the placenta itself. Our results suggest that the life of the placenta may represent a fractal of the life of the organism in its attempt to manage and prepare for the vicissitudes of life. With further longitudinal follow-up, it may become possible to use genetic analysis of the placenta to anticipate the risks for stress-related developmental psychopathology and identify children and families who might benefit from psychosocial prevention programs.

## Supporting information

S1 Table Correlations of all variables. (DOCX)

S2 Table Fit statistics for latent classes determining normative prenatal stress. (DOCX)

S3 Table The 40 candidate genes. (DOCX)

S1 Data (XLS)
