# Supplementary material for: Moderate prenatal stress may buffer the impact of Superstorm Sandy on placental genes: Stress in Pregnancy (SIP) Study
Source: PLoS One. 2020 Jan 29;15(1):e0226605. doi: 10.1371/journal.pone.0226605 (PMC6988921; doi:10.1371/journal.pone.0226605)
Supplement: S1 Table — (DOCX) [file pone.0226605.s001.docx]

S1 Table

*Correlations of All Variables.*

|  | 1 | 2 | 3 | 4 | 5 | 6 | 7 | 8 | 9 |
| --- | --- | --- | --- | --- | --- | --- | --- | --- | --- |
| 1. EPDS | 1 |  |  |  |  |  |  |  |  |
| 2. PRAQ | 0.48 | 1 |  |  |  |  |  |  |  |
| 3. PSS | 0.55 | 0.30 | 1 |  |  |  |  |  |  |
| 4. STAI-S | 0.67 | 0.38 | 0.59 | 1 |  |  |  |  |  |
| 5. STAI-T | 0.74 | 0.48 | 0.66 | 0.83 | 1 |  |  |  |  |
| 6. Neg Events | 0.41 | 0.19 | 0.33 | 0.31 | 0.35 | 1 |  |  |  |
| 7. CDKL5 | -0.03 | -0.06 | 0.07 | 0.01 | 0.04 | 0.13 | 1 |  |  |
| 8. CFL1 | -0.05 | -0.10 | 0.08 | 0.02 | 0.03 | 0.17 | 0.57 | 1 |  |
| 9. CRHBP | -0.05 | -0.09 | -0.04 | -0.01 | -0.07 | -0.01 | 0.19 | 0.17 | 1 |
| 10. DBH | 0.01 | -0.09 | -0.04 | -0.01 | 0.03 | 0.09 | 0.20 | 0.23 | 0.29 |
| 11. DYRK1A | 0.03 | 0.00 | 0.12 | 0.14 | 0.11 | 0.14 | 0.59 | 0.54 | -0.07 |
| 12. FOXP1 | 0.00 | -0.11 | 0.00 | 0.04 | 0.04 | 0.08 | 0.26 | 0.28 | 0.26 |
| 13. HSD11B2 | 0.02 | 0.03 | 0.16 | 0.09 | 0.10 | 0.17 | 0.51 | 0.50 | -0.07 |
| 14. MAOA | -0.02 | -0.02 | 0.12 | 0.06 | 0.08 | 0.17 | 0.48 | 0.56 | -0.02 |
| 15. MAOB | -0.03 | 0.01 | 0.03 | 0.03 | -0.02 | -0.07 | -0.06 | 0.02 | -0.25 |
| 16. MECP2 | 0.06 | -0.04 | 0.05 | 0.15 | 0.12 | 0.16 | 0.57 | 0.58 | 0.16 |
| 17. NCOR1 | -0.01 | -0.04 | 0.07 | 0.08 | 0.10 | 0.13 | 0.68 | 0.63 | 0.24 |
| 18. NR3C1 | -0.05 | -0.14 | -0.04 | 0.04 | 0.05 | 0.11 | 0.56 | 0.47 | 0.39 |
| 19. NR3C2 | 0.01 | -0.01 | 0.04 | 0.12 | 0.12 | 0.19 | 0.53 | 0.35 | 0.10 |
| 20. ZNF507 | 0.07 | 0.08 | 0.11 | 0.12 | 0.10 | 0.11 | 0.32 | 0.30 | -0.22 |
| 21. Delivery Method | 0.01 | -0.01 | -0.04 | 0.04 | 0.04 | 0.07 | 0.01 | -0.02 | 0.16 |
| 22. Inf Sex | -0.08 | -0.05 | 0.06 | -0.07 | -0.01 | -0.01 | -0.02 | 0.05 | -0.05 |
| 23. Mo Ed | -0.01 | 0.08 | -0.08 | 0.00 | -0.01 | -0.08 | -0.02 | -0.04 | 0.11 |

|  | 10 | 11 | 12 | 13 | 14 | 15 | 16 | 17 |
| --- | --- | --- | --- | --- | --- | --- | --- | --- |
| 1. EPDS |  |  |  |  |  |  |  |  |
| 2. PRAQ |  |  |  |  |  |  |  |  |
| 3. PSS |  |  |  |  |  |  |  |  |
| 4. STAI-S |  |  |  |  |  |  |  |  |
| 5. STAI-T |  |  |  |  |  |  |  |  |
| 6. Neg Events |  |  |  |  |  |  |  |  |
| 7. CDKL5 |  |  |  |  |  |  |  |  |
| 8. CFL1 |  |  |  |  |  |  |  |  |
| 9. CRHBP |  |  |  |  |  |  |  |  |
| 10. DBH | 1 |  |  |  |  |  |  |  |
| 11. DYRK1A | 0.02 | 1 |  |  |  |  |  |  |
| 12. FOXP1 | 0.16 | 0.13 | 1 |  |  |  |  |  |
| 13. HSD11B2 | -0.03 | 0.70 | 0.01 | 1 |  |  |  |  |
| 14. MAOA | 0.01 | 0.72 | 0.03 | 0.87 | 1 |  |  |  |
| 15. MAOB | -0.16 | 0.06 | -0.05 | 0.03 | 0.08 | 1 |  |  |
| 16. MECP2 | 0.23 | 0.66 | 0.44 | 0.28 | 0.33 | 0.08 | 1 |  |
| 17. NCOR1 | 0.21 | 0.74 | 0.32 | 0.35 | 0.46 | -0.06 | 0.86 | 1 |
| 18. NR3C1 | 0.48 | 0.38 | 0.50 | 0.13 | 0.20 | -0.16 | 0.71 | 0.74 |
| 19. NR3C2 | 0.11 | 0.65 | 0.14 | 0.40 | 0.47 | -0.12 | 0.56 | 0.70 |
| 20. ZNF507 | -0.08 | 0.69 | 0.19 | 0.71 | 0.62 | 0.17 | 0.30 | 0.22 |
| 21. Delivery Method | 0.04 | 0.01 | 0.08 | -0.01 | 0.00 | -0.03 | 0.07 | 0.08 |
| 22. Inf Sex | 0.07 | 0.02 | -0.05 | 0.09 | 0.10 | 0.03 | -0.01 | -0.02 |
| 23. Mo. Edu | -0.02 | -0.03 | 0.06 | -0.10 | -0.09 | -0.01 | 0.03 | 0.00 |

|  | 18 | 19 | 20 | 21 | 22 | 23 |
| --- | --- | --- | --- | --- | --- | --- |
| 1. EPDS |  |  |  |  |  |  |
| 2. PRAQ |  |  |  |  |  |  |
| 3. PSS |  |  |  |  |  |  |
| 4. STAI-S |  |  |  |  |  |  |
| 5. STAI-T |  |  |  |  |  |  |
| 6. Neg Events |  |  |  |  |  |  |
| 7. CDKL5 |  |  |  |  |  |  |
| 8. CFL1 |  |  |  |  |  |  |
| 9. CRHBP |  |  |  |  |  |  |
| 10. DBH |  |  |  |  |  |  |
| 11. DYRK1A |  |  |  |  |  |  |
| 12. FOXP1 |  |  |  |  |  |  |
| 13. HSD11B2 |  |  |  |  |  |  |
| 14. MAOA |  |  |  |  |  |  |
| 15. MAOB |  |  |  |  |  |  |
| 16. MECP2 |  |  |  |  |  |  |
| 17. NCOR1 |  |  |  |  |  |  |
| 18. NR3C1 | 1 |  |  |  |  |  |
| 19. NR3C2 | 0.50 | 1 |  |  |  |  |
| 20. ZNF507 | 0.08 | 0.23 | 1 |  |  |  |
| 21. Delivery Method | 0.16 | 0.00 | 0.01 | 1 |  |  |
| 22. Inf Sex | 0.03 | 0.00 | 0.06 | -0.10 | 1 |  |
| 23. Mo. Edu | 0.02 | -0.01 | -0.07 | 0.09 | -0.05 | 1 |

NB. Neg event = negative life events; Inf Sex = infant sex; Mo. Edu = maternal education
