# Supplementary material for: Moderate prenatal stress may buffer the impact of Superstorm Sandy on placental genes: Stress in Pregnancy (SIP) Study
Source: PLoS One. 2020 Jan 29;15(1):e0226605. doi: 10.1371/journal.pone.0226605 (PMC6988921; doi:10.1371/journal.pone.0226605)
Supplement: S2 Table — (DOCX) [file pone.0226605.s002.docx]

S2 Table *Fit statistics for Latent Classes Determining Normative Prenatal Stress.*

| Class solution | BIC | ABIC | L-M-R  p-value | Entropy | Percentages of class counts |
| --- | --- | --- | --- | --- | --- |
| 2 Classes | 10565.36 | 10505.11 | .002 | .838 | 61.9%, 38.1% |
| 3 Classes | 10346.75 | 10264.29 | .018 | .871 | 43.6%, 38.3%, 18.2% |
| 4 Classes | 10322.17 | 10217.51 | .344 | .818 | 34.7%, 28.7%, 22.4%, 14.2% |

Note: Entropy values are acceptable for all models. BIC, Bayesian Information Criterion. ABIC, adjusted BIC. L-M-R, Lo-Mendell-Rubin test.
