# Supplementary material for: Moderate prenatal stress may buffer the impact of Superstorm Sandy on placental genes: Stress in Pregnancy (SIP) Study
Source: PLoS One. 2020 Jan 29;15(1):e0226605. doi: 10.1371/journal.pone.0226605 (PMC6988921; doi:10.1371/journal.pone.0226605)
Supplement: S3 Table — (DOCX) [file pone.0226605.s003.docx]

S3 Table *The 40 Candidate Genes.*

|  | **HPA-axis Function** | **Neurodevelopment** |
| --- | --- | --- |
| 27 Expressed | *CFL1, CREB1, CREBBP, DYRK1A, HSD11Β1, HSD11Β2, NCOR1, NCOR2, AVPR1B, CRHBP, NR3C1, NR3C2, NR4A1, POMC* | *ADRA2A, CDKL5, DBH, FOXP1, HTR1B, MAOA, MAOB, MECP2, PON3, SNAP25, SRD5A3, ZNF507, ZNHIT6* |
| 13 Unexpressed | *AVP, AVPR1A, CRH2R, CRH1R, UCN, UCN3* | *ADRA1A, ADRA1B, DAOA, KIRREL3, NRG1, TPH1, TPH2* |

Expressed is defined as genes for which more than 50% of the sample fell above the limit of detection. Unexpressed as 50% or less.
